# Comparative Transcriptome Analysis Reveals the Effect of the DHN Melanin Biosynthesis Pathway on the Appressorium Turgor Pressure of the Poplar Anthracnose-Causing Fungus *Colletotrichum gloeosporioides*

**DOI:** 10.3390/ijms24087411

**Published:** 2023-04-18

**Authors:** Xinyu Qin, Chengming Tian, Fanli Meng

**Affiliations:** 1 The Key Laboratory for Silviculture and Conservation of Ministry of Education, College of Forestry, Beijing Forestry University, Beijing 100083, China; 2 Beijing Key Laboratory for Forest Pest Control, College of Forestry, Beijing Forestry University, Beijing 100083, China

**Keywords:** *Colletotrichum gloeosporioides*, appressorium, turgor generation, DHN melanin biosynthesis

## Abstract

Anthracnose of poplar caused by *Colletotrichum gloeosporioides* is a leaf disease that seriously affects poplar growth. The pathogen invades the host in the form of adherent cells, which generate turgor pressure through the metabolism of intracellular substances prior to penetrating the epidermis of poplar leaves. In this study, the expansion-related pressure of the mature appressorium of the wild-type *C. gloeosporioides* was approximately 13.02 ± 1.54 MPa at 12 h, whereas it was 7.34 ± 1.23 MPa and 9.34 ± 2.22 MPa in the melanin synthesis-related gene knockout mutants Δ*CgCmr1* and Δ*CgPks1*, respectively. The *CgCmr1* and *CgPks1* genes were highly expressed at 12 h in the wild-type control, implying that the DHN melanin biosynthesis pathway may play an important role in the mature appressorium stage. The transcriptome sequencing analysis indicated that the upregulated melanin biosynthesis genes in *C. gloeosporioides*, such as *CgScd1*, *CgAyg1*, *CgThr1*, *CgThr2*, and *CgLac1*, are involved in specific KEGG pathways (i.e., fatty acid biosynthesis, fatty acid metabolism, and biotin metabolism). Therefore, we speculate that the melanin synthesis-related genes and fatty acid metabolism pathway genes contribute to the regulation of the turgor pressure in the mature *C. gloeosporioides* appressorium, ultimately leading to the formation of infection pegs that enter plant tissues. These observations may reflect the co-evolution of *C. gloeosporioides* and its host.

## 1. Introduction

Poplar anthracnose is caused by *Colletotrichum gloeosporioides* [1] and is an extremely destructive disease. Among the species in the genus *Colletotrichum*, *C. gloeosporioides* is the pathogen with the widest host range and is responsible for causing the most severe damage. In addition to infecting poplar trees, *C. gloeosporioides* can also infect walnut, mango, rubber, and other economically valuable forest tree species (occurrence area exceeding 60,000 hectares), as well as a variety of cultivated plants, resulting in rotted leaves, branches, or fruits and substantial losses in agriculture and forestry industries [2,3]. Poplar trees in Ningxia, Beijing, Henan, Hebei, Shanxi, and other regions in China have been damaged to varying degrees. Wind, rain, and insects are the main vectors of *C. gloeosporioides*, with wind serving as the primary vector for the infection of poplar trees.

When conidia attach to the surface of poplar leaves, they germinate and form a bud tube. The tip of the bud tube expands to form an initial appressorium. The metabolism of substances in the appressorium results in swelling. When the swelling is converted to a mechanical force, the base of the appressorium produces fine infection pegs that penetrate the epidermis of poplar leaves, leading to the successful invasion of the underlying cells. The formation of a functional appressorium is critical for the interaction between *C. gloeosporioides* and different resistant hosts. Therefore, the regulated metabolism of specific substances in the adherent cells of *C. gloeosporioides* is crucial for appressorium formation and the completion of the infection process [4,5,6].

The swelling of appressoria produced by pathogenic fungi has been investigated. More specifically, *Magnaporthe oryzae*, which is a model pathogenic fungus, has been thoroughly studied in terms of its infection structure (appressorium) function and the mechanism underlying its swelling. There are two factors mediating the increase in pressure of the appressorium of the rice blast fungus. First, 1,8-dihydroxynaphthalene (1,8-DHN) melanin functions as an effective permeability barrier to increase the cell solute concentration. Second, whether the osmotic pressure in the cytoplasm can increase sufficiently depends on the characteristics of cytosol [7]. Previous research has confirmed that melanin may serve as a rigid structural barrier that prevents solute efflux, which is crucial for the formation of turgor pressure [8,9,10]. However, there is relatively little melanin deposited in the appressorial cell wall adhering to the plant surface, and a pore eventually forms that allows small molecules (e.g., water) to pass through, but not organic molecules (e.g., glycerol) [11]. The infection peg extending from this aperture penetrates the epidermal cells because of the considerable swelling-related pressure and then enters the adjacent cells through plasmodesmata, ultimately resulting in the widespread distribution of the pathogenic fungus, which proceeds to the necrotic growth stage and to the development of disease symptoms [3]. The highest turgor pressure of the attached cells can exceed 8.0 Mpa.

Relevant studies have been conducted to clarify the molecular mechanism underlying *C. gloeosporioides* appressorium formation and the subsequent infection of plant hosts, but they have been relatively rare and mainly focused on the functional annotation of genes involved in appressorium formation and regulation [12,13,14]. Previous studies on poplar anthracnose have shown that the *C. gloeosporioides* adherent cells must metabolize certain materials and the turgor pressure needs to increase in order to facilitate entry into the host tissues. During their maturation, the translucent adherent cells continuously accumulate melanin to enhance the strength of their cell wall, enabling them to withstand the increase in turgor pressure [15,16]. The *C. gloeosporioides* genes encoding the regulators of melanin synthesis have been identified. Moreover, mutants in which DHN melanin synthesis genes are silenced have been generated [16]. A recent study indicated that among the melanin synthesis-related genes in *C. gloeosporioides*, the deletion of *CgPks1* will lead to a lack of melanin production, whereas the deletion of *CgCmr1* results in delayed and limited melanin synthesis in the appressorium [16]. In addition, Δ*CgCmr1* and Δ*CgPks1* mutants were also found to be sensitive to H_2_O_2_ oxidative stress, but not to other abiotic stresses, which suggests that *CgCmr1* and *CgPks1* may play a key role in DHN melanin biosynthesis. However, the results of Wang et al. [16] did not reveal the effect of the DHN melanin biosynthesis pathway on appressorium turgor pressure.

In this study, we analyzed the *C. gloeosporioides* transcriptome to reveal differences in the expression profiles of the genes associated with the DHN melanin biosynthesis pathway during the increase in turgor pressure in the appressorium.

## 2. Results

### 2.1. Appressorium Morphology in Different Developmental Stages

**Figure 1:**
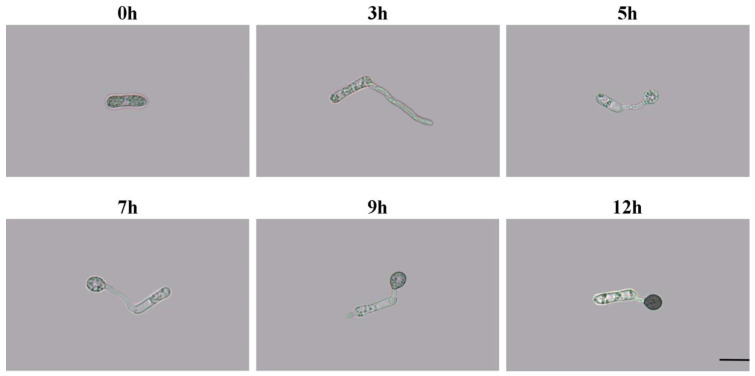
Morphological characteristics of the *Colletotrichum gloeosporioides* appressorium on a hydrophobic surface at different time points (scale = 10 μm).

Appressorium morphogenesis was analyzed at the following time points: 0 h (conidia), 3 h (germ tube), 5 h (initial appressorium), 7 h, and 9 h (gradually maturing appressorium), and 12 h (mature appressorium) (Figure 1).

### 2.2. Changes in the Appressorium Turgor Pressure in the Wild-Type Control and Mutants and CgCmr1 and CgPks1 Expression in the Wild-Type Control at Different Time Points

**Figure 2:**
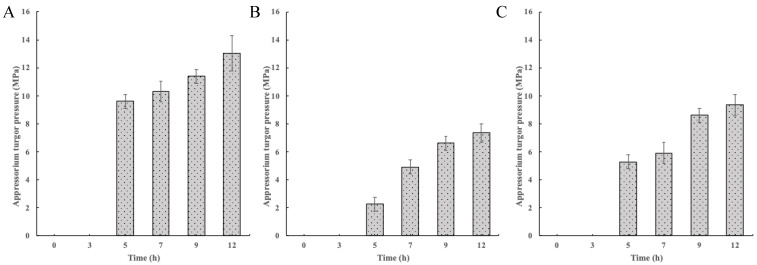
Appressorium turgor pressure for the wild-type (WT) control (**A**) and Δ*CgCmr1* (**B**) and Δ*CgPks1* (**C**) mutants at different time points.

**Figure 3:**
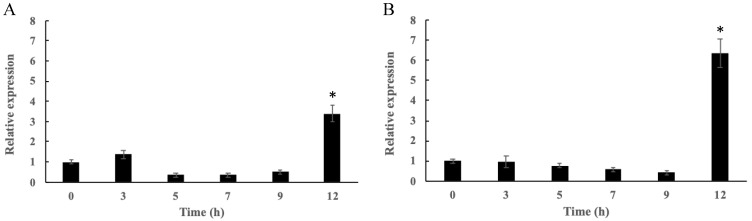
Relative expression of *CgCmr1* (**A**) and *CgPks1* (**B**) in the wild-type control at different time points. * Significant differences among time points as determined by the *t*-test (*p* < 0.05).

The analysis of the adherent cell turgor pressure of the *C. gloeosporioides* WT control and two mutants revealed an increase in the adherent cell turgor pressure of the WT control over time. More specifically, it was 9.60 ± 1.04 MPa at 5 h, 10.29 ± 0.53 MPa at 7 h, 11.38 ± 0.65 MPa at 9 h, and 13.02 ± 1.54 MPa at 12 h. The adherent cell turgor pressure of Δ*CgCmr1* was 2.26 ± 0.58 MPa at 5 h, 4.91 ± 0.98 MPa at 7 h, 6.6 ± 0.67 MPa at 9 h, and 7.34 ± 1.23 MPa at 12 h. The adherent cell turgor pressure of Δ*CgPks1* was 5.26 ± 1.31 MPa at 5 h, 5.91 ± 0.55 MPa at 7 h, 8.60 ± 1.00 MPa at 9 h, and 9.34 ± 2.22 MPa at 12 h (Figure 2). The qRT-PCR analysis showed that *CgCmr1* and *CgPks1* were highly expressed in the WT control at 12 h (Figure 3), indicative of the importance of the DHN melanin biosynthesis pathway in the mature appressorium stage.

### 2.3. Transcriptome Sequencing and Data Assembly

On the basis of the above-mentioned results, we sequenced the transcriptome of the conidia (0 h) and mature appressorium (12 h) of the *C. gloeosporioides* WT strain to explore the effect of the DHN melanin biosynthesis pathway on the appressorium turgor pressure. The raw reads generated for the *C. gloeosporioides* CgWT0h and CgWT12h samples comprised 7,534,480,292 and 6,803,953,388 bp, respectively (Table 1). The adapter sequences, reads with ambiguous nucleotides, and low-quality reads were eliminated to obtain the clean reads for the CgWT0h and CgWT12h samples. More than 85% of the reads in all six samples had a quality score exceeding Q30 and the Q30 percentage for all samples was not less than 93.94% (Table 1), reflecting the high quality of the sequencing data. Additionally, more than 5.53 Gb clean bases were obtained per library.

### 2.4. Differentially Expressed Genes among the Transcriptomes

**Figure 4:**
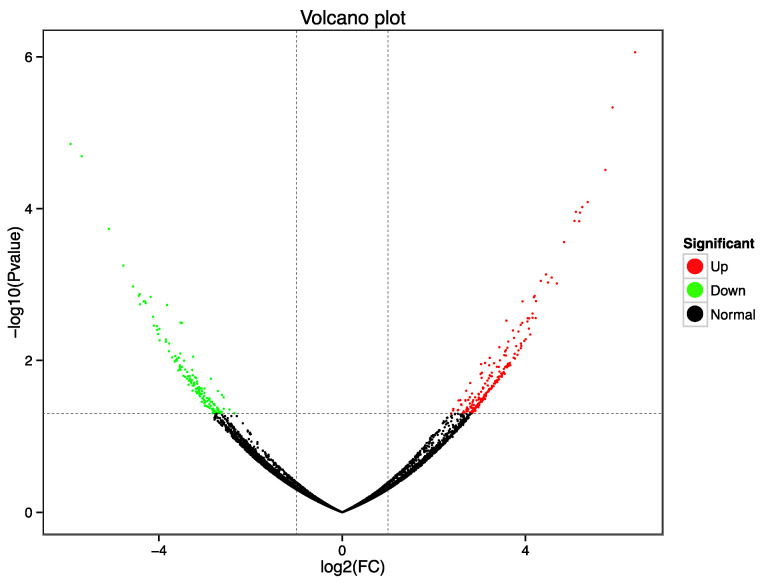
Number of DEGs in the CgWT12h sample. Red and green indicate upregulated and downregulated expression, respectively.

The CgWT0h and CgWT12h transcriptome profiles were compared to identify the DEGs. Of the 380 DEGs between CgWT0h and CgWT12h, 231 and 149 were upregulated and downregulated, respectively. The DEG distribution is presented in Figure 4; the significant DEGs were identified on the basis of an adjusted fold-change ≥2 and a corrected *p* < 0.01. The GO molecular function category included 10 upregulated DEGs (CgWT12h) that were assigned to subcategories, among which five may be involved in metabolic activities regulating the generation of turgor pressure (i.e., lipid transport and metabolism activity; energy production and conversion activity; secondary metabolite biosynthesis, transport, and catabolism activity; posttranslational modification activity; and amino acid transport and metabolism activity) (Table 2). In addition, the 10 downregulated DEGs (CgWT12h) included genes related to carbohydrate transport and metabolism activity, inorganic ion transport and metabolism activity, short chain dehydrogenase activity, and glycosyl hydrolase activity (Table 2).

### 2.5. GO Enrichment Analysis of the DEGs

**Figure 5:**
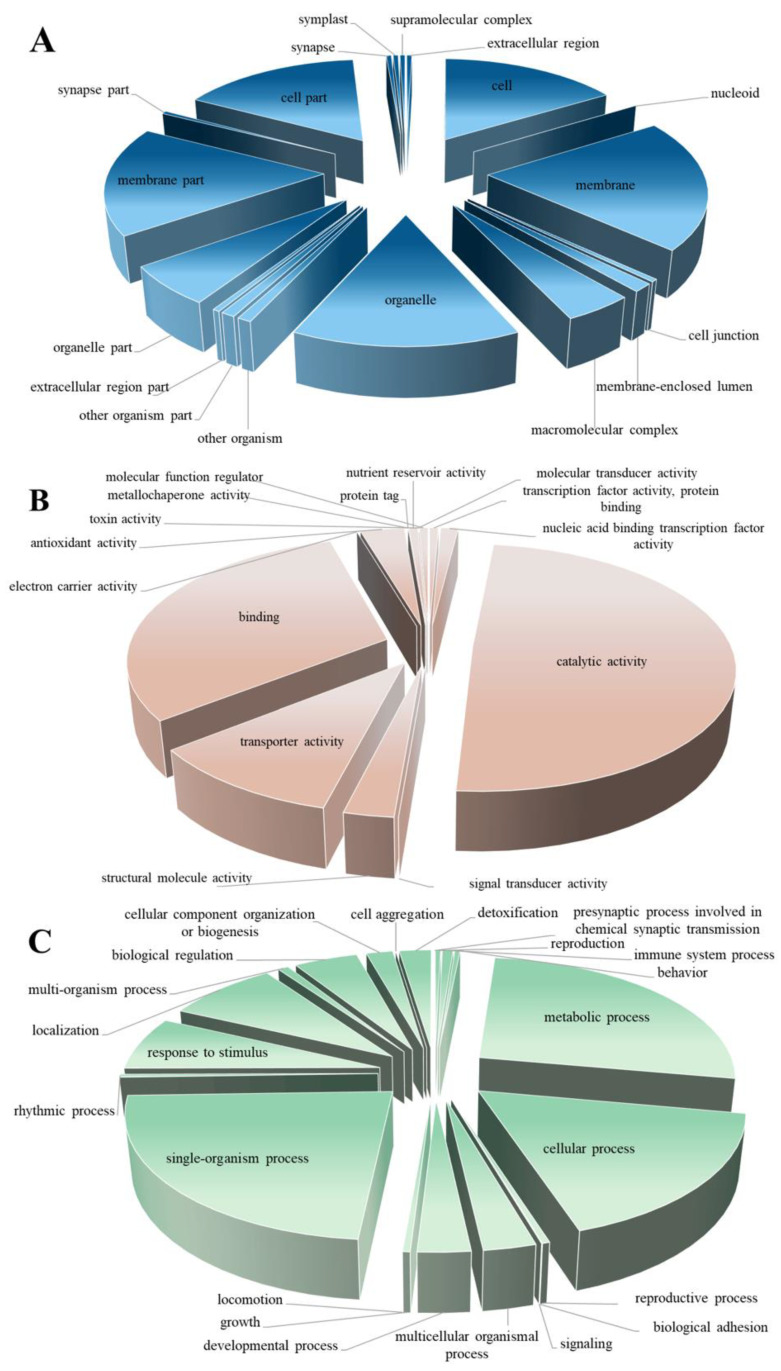
GO term annotation of the DEGs in the CgWT12h transcriptome. The DEGs in the CgWT12h sample were assigned GO terms from the following three main categories: cellular component (**A**), molecular function (**B**), and biological process (**C**).

A GO enrichment analysis was performed to functionally annotate and classify the DEGs. The upregulated and downregulated genes were assigned GO IDs and categories (Figure 5). The main GO categories were the cellular component, molecular function, and biological process. For the CgWT12h sample, the GO analysis revealed the following. In the biological process category, 25.81% and 21.77% of the DEGs were annotated with the metabolic process (GO:0008152) and cellular metabolic process (GO:0044237), respectively. In the molecular function category, 20.93% and 18.02% of the DEGs were annotated with the catalytic activity (GO:0003824) and oxidoreductase activity (GO:0016491), respectively. In the cellular component category, 33.93% and 27.68% of the DEGs were annotated with the cell (GO:0005623) and intracellular organelle (GO:0043229), respectively.

### 2.6. Enriched KEGG Pathways among the DEGs

A total of 67 KEGG pathways were enriched among 136 DEGs in the CgWT12h sample (Table 3). The major enriched metabolic pathways included the following: carbon metabolism; biosynthesis of amino acids; tryptophan metabolism; glycine, serine, and threonine metabolism; fatty acid metabolism; phenylpropanoid biosynthesis; tyrosine metabolism; arginine and proline metabolism; glycerophospholipid metabolism; and carotenoid biosynthesis.

Both *CgCmr1* and *CgPks1* encode regulators of melanin biosynthesis in *C. gloeosporioides*. In the current study, *CgScd1* (EVM0006990), *CgAyg1* (EVM0012797), *CgThr1* (EVM0003085), *CgThr2* (EVM0013226), and *CgLac1* (EVM0011157) were detected as upregulated genes associated with the KEGG pathways fatty acid biosynthesis (ko00061; Supplementary Figure S1), fatty acid metabolism (ko01212; Supplementary Figure S2), and biotin metabolism (ko00780; Supplementary Figure S3).

### 2.7. Verification of Gene Expression by qRT-PCR

**Figure 6:**
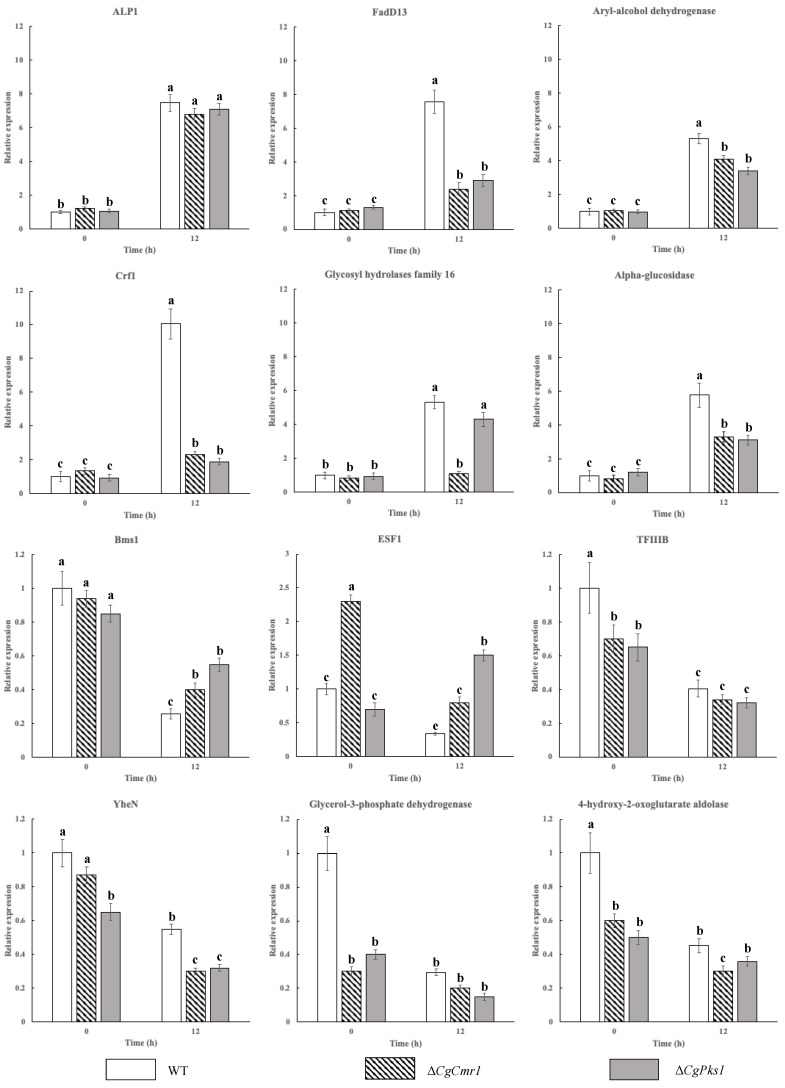
Results of the qRT-PCR analysis performed to validate key candidate DEGs in the wild-type (WT) control and Δ*CgCmr1* and Δ*CgPks1* mutants at 0 and 12 h. The qRT-PCR data represent the mean of three replicates. Bars indicate the standard error. Different letters indicate significant differences among treatments, as determined by Duncan’s test (*p* < 0.05).

To verify the accuracy of the transcriptome data, we selected a few potentially key DEGs identified in the WT, Δ*CgCmr1*, and Δ*CgPks1* transcriptomes at 0 and 12 h for qRT-PCR analysis. More specifically, DEGs encoding proteins contributing to fatty acid biosynthesis, fatty acid metabolism, and biotin metabolism were analyzed. The qRT-PCR data were consistent with the corresponding RNA-seq data for the 12 selected genes encoding the following proteins: long-chain-fatty-acid—CoA ligase (FadD13), aryl-alcohol dehydrogenase, transcription factor Crf1, glycosyl hydrolase family 16 member, alpha-glucosidase, alkali-sensitive linkage protein (ALP1), polysaccharide deacetylase (YheN), glycerol-3-phosphate dehydrogenase, 4-hydroxy-2-oxoglutarate aldolase, ribosome biogenesis protein (Bms1), pre-rRNA-processing protein (ESF1), and transcription factor (TFIIIB). Thus, the qRT-PCR analysis confirmed the RNA-seq data were reliable and accurate (Figure 6). Furthermore, the expression levels of the genes encoding FadD13, Crf1, and alpha-glucosidase, which influence fatty acid biosynthesis and fatty acid metabolism, were significantly downregulated at 12 h in the Δ*CgCmr1* and Δ*CgPks1* mutants.

## 3. Discussion

When *C. gloeosporioides* infects poplar trees, its conidia attach to the leaf surface and germinate to form a bud tube. The tip of the bud tube expands to form an initial appressorium. The metabolic activities in the appressorium lead to swelling and an increase in turgor pressure. Next, infection pegs at the appressorium base penetrate the poplar leaf epidermis and invade the underlying cells [4,5,6]. A functional appressorium mediates *C. gloeosporioides*–host interactions.

After the *C. gloeosporioides* appressorium forms, certain materials are metabolized and the appressorium swells, thereby enabling it to invade the host tissue. During its maturation, the translucent appressorium accumulates melanin to strengthen its cell wall, which is necessary because of the increased pressure due to swelling [16]. By measuring the expansion-related pressure as the *C. gloeosporioides* appressorium morphology changed, we revealed that the turgor pressure of the mature appressorium (i.e., at 12 h) was greater for the WT *C. gloeosporioides* (13.02 ± 1.54 MPa) than for the Δ*CgCmr1* (7.34 ± 1.23 MPa) and Δ*CgPks1* (9.34 ± 2.22 MPa) mutants.

The qRT-PCR analysis showed that *CgCmr1* and *CgPks1* were expressed at high levels in the WT control at 12 h, suggestive of the importance of the DHN melanin biosynthesis pathway in the mature appressorium. We constructed transcriptome sequencing libraries using the total RNA extracted from the *C. gloeosporioides* samples collected from the GelBond membrane surface at 0 h (conidia) and 12 h (mature appressorium). The enriched KEGG pathways among the upregulated *C. gloeosporioides* melanin biosynthesis-related genes, such as *CgScd1* (EVM0006990), *CgAyg1* (EVM0012797), *CgThr1* (EVM0003085), *CgThr2* (EVM0013226), and *CgLac1* (EVM0011157), were fatty acid biosynthesis (ko00061), fatty acid metabolism (ko01212), and biotin metabolism (ko00780). Polyketide synthase is one of the initial enzymes in the DHN melanin biosynthesis pathway [17]. Mutations to *Pks1* genes result in a complete lack of melanin biosynthesis in many fungi, including *Verticillium dahliae* [18], *Botrytis cinerea* [19], *Colletotrichum falcatum* [20], and *Colletotrichum orbiculare* [21]. The *Cmr1* transcription factor gene, which is involved in DHN melanin biosynthesis, has also been widely studied in several fungi, including *M. oryzae* (*Pig1*) [22] and *V. dahliae* (*VdCmr1*) [23]. Moreover, Wang et al. [16] found that the deletion of *CgPks1* will lead to a lack of melanin production, whereas the deletion of *CgCmr1* results in delayed and limited melanin synthesis in the appressorium, which suggests that *CgCmr1* and *CgPks1* may play a key role in DHN melanin biosynthesis. Based on the transcriptome data of this study, we speculate that the melanin synthesis-related genes and fatty acid metabolism pathway genes in the mature *C. gloeosporioides* appressorium encode proteins that coordinately modulate the turgor pressure, which enables the formation of the infection pegs needed to invade host tissues.

It has been reported that the upstream transcription factors may regulate DHN melanin biosynthesis [16]. The results of the current study show that the expression of the transcription factor gene *Crf1* was upregulated at 12 h (mature appressorium stage) in the WT strain, but downregulated in the Δ*CgCmr1* and Δ*CgPks1* mutants. The transcription factors downstream of cAMP–PKA (e.g., Crf1) can regulate multiple metabolic pathways in *M. oryzae*, which may affect appressorium formation. The silencing of *Crf1* will delay the transfer and transformation of lipids and carbon materials in the appressorium, resulting in a decrease in the turgor pressure and the abnormal formation of infection pegs [24]. The transcription factor Crf1 has a basic helix–loop–helix domain. In *M. oryzae*, the deletion or mutation of *Crf1* may lead to the defective use of lipids, glycerol, arabinose, and other substances, some of which are involved in lipolysis, as well as the downregulated expression of genes associated with oxidation, glucose metabolism, gluconeogenesis, and glycerol metabolism, which decreases the turgor pressure in the adherent cells [25]. Hence, we speculate that the silencing of melanin synthesis genes may inhibit intracellular signaling and decrease the expression of specific genes, including *CgCrf1*, resulting in lipolysis. Moreover, the expression levels of important genes contributing to oxidation, glucose metabolism, gluconeogenesis, and glycerol metabolism were downregulated, which affected the appressorium turgor pressure.

The infection of poplar by *C. gloeosporioides* and the infection of rice by the rice blast fungus involve a similar process. In both cases, conidia germinate and produce a bud tube and appressorium. However, because of the differences in physiological structures and the growth environment at the leaf surface of woody and herbaceous plants, the formation of a mature rice blast fungus appressorium (turgor pressure of approximately 8 MPa) following the germination of conidia requires 24 h [26,27,28], whereas only 12 h is needed for the formation of the mature *C. gloeosporioides* appressorium (turgor pressure of approximately 13 MPa). The change in the turgor pressure during appressorium formation is primarily due to the regulated metabolism of specific materials. Accordingly, during the development of a mature appressorium, there are dynamic changes to metabolic products in the cell. Although the overall process mediating the formation of different types of appressorium may be similar, there are also specific differences. For example, there is a clear species-specificity in the compounds involved in the swelling of the appressorium. The rapid swelling of the *C. gloeosporioides* appressorium may be related to a particular molecular mechanism regulating the formation of turgor pressure via the metabolism of compounds in *C. gloeosporioides* cells adhering to the host, suggestive of the co-evolution of *C. gloeosporioides* and its host [29].

In conclusion, the findings of our study show that the DHN melanin biosynthesis pathway may play an important role in the appressorium turgor pressure generation of *C. gloeosporioides.* Meanwhile, the melanin synthesis-related genes and fatty acid metabolism pathway genes may also contribute to the regulation of turgor pressure in the mature *C. gloeosporioides* appressorium, which may provide the foundation for future research conducted to verify the functions of *C. gloeosporioides* genes encoding proteins modulating the turgor pressure in developing appressoria prior to the invasion of poplar tissues.

## 4. Materials and Methods

### 4.1. Materials

The wild-type (WT) *C. gloeosporioides* strain CFCC80308 used in this study was isolated from *Populus* × *beijingensis* in Beijing, China. The Δ*CgCmr1* and Δ*CgPks1* mutants were generated in a previous study [16]. All of the strains were cultured on potato dextrose agar (PDA, Oxoid, UK) medium in plates at 25 °C.

### 4.2. Determination of the Turgor Pressure in WT, ΔCgCmr1, and ΔCgPks1 Appressoria at Different Time Points

The conidia of the WT and mutant strains were harvested from 5-day-old cultures growing on PDA medium in plates and then washed three times in sterile water. The conidia were maintained on the artificial hydrophobic surface of the GelBond membrane (LONZA, Basel, Switzerland) at 25 °C in darkness to induce appressorium formation. To promote germination, the conidia were diluted in sterile water (2 × 10^5^ conidia/mL) on the artificial hydrophobic surface of the GelBond membrane. The GelBond membrane surface was scraped using a cell shovel at 3, 5, 7, and 12 h for the examination using a light microscope. The analysis was performed using different PEG-2000 concentrations. Specifically, the GelBond membrane containing conidia was transferred to a glass slide, after which the water was removed and PEG-2000 solutions at different concentrations were added dropwise. Each treatment was repeated five times. After 10 min, statistical counts were performed using the light microscope. The osmotic pressure of the solution that caused 50% of the appressoria to collapse was recorded as the estimated value for the appressorium.

### 4.3. Total RNA Isolation and cDNA Synthesis

The samples were collected from the GelBond membrane surface at 0 h (conidia) and 12 h (mature appressorium). They were immediately frozen in liquid nitrogen and then stored at −80 °C. The total RNA was extracted from the treated *C. gloeosporioides* samples (see previous section) using the TRIzol reagent (Invitrogen, Carlsbad, CA, USA) according to the manufacturer’s instructions. The RNA integrity (minimum RNA integrity number of 8) was confirmed using the Agilent 2100 Bioanalyzer (Agilent, Mainz, Germany). At least 3 μg total RNA was obtained from each sample. For the transcriptome analysis, samples were prepared using reagents from an Illumina TruSeq^TM^ RNA sample preparation Kit (San Diego, CA, USA) following the manufacturer’s instructions. Briefly, mRNA was purified from the total RNA samples using oligo-(dT) magnetic beads and then fragmented into short sequences in a fragmentation buffer. The obtained mRNA fragments were used as the template to synthesize the first and second cDNA strands, which were synthesized using a SuperScript double-stranded cDNA synthesis kit (Invitrogen, Carlsbad, CA, USA) with random hexamer primers (Illumina, San Diego, CA, USA). The cDNA was purified using AMPure XP beads. Synthesized cDNAs were subjected to end-repair, phosphorylation, and “A” base addition according to Illumina’s library construction protocol. This was followed by a PCR amplification to complete the construction of the sequencing library. A quantitative real-time polymerase chain reaction (qRT-PCR) analysis was performed to accurately quantify the effective concentration of the library and to ensure its quality was sufficient for sequencing.

### 4.4. Sequencing and Assembly of the C. gloeosporioides Transcriptome

Three replicates were prepared for each sample (six cDNA libraries) and the average data for the raw reads were recorded. The six cDNA libraries were sequenced using the Illumina HiSeq 2000 platform at the Beijing Genome Institute (Shenzhen, China). The retained reads were aligned to the *C. gloeosporioides* genome to identify new genes. In addition, the retained reads were aligned to the reference transcriptome and the gene expression was stratified and indexed using the Hierarchical Indexing for Spliced Alignment of Transcripts 2 (HISAT2) software (v.2.0.4) [30]. StringTie was used to analyze the data (reads with a mapping quality greater than 30) [31], resulting in a standardized count for each gene (expressed in terms of fragments per kilobase per million mapped fragments). Raw Illumina reads were trimmed and filtered to remove low-quality reads using Trimmomatic (v.0.33). The filtered reads were aligned to the reference genome using TopHat (v.2.0.13). The Htseq-count function of HTSeq (v.0.6.1) was used to calculate the gene counts. Because the Htseq-count excluded the mapped reads generated by TopHat with more than one reported alignment, only the unique mapped reads were used for the downstream analysis. The differentially expressed genes (DEGs) among the samples were identified on the basis of negative binomial distribution using the DESeq R package (1.10.1) [32]. The resulting *p*-values were adjusted according to the Benjamini–Hochberg method and then set (adjusted *p* < 0.05) as the threshold for detecting significant differences in expression [33]. Translated transcript sequences were used as queries for the Blastx (http://blast.ncbi.nlm.nih.gov/Blast.cgi, accessed on 13 October 2022) search of the nonredundant (nr), Swiss-Prot, and Kyoto Encyclopedia of Genes and Genomes (KEGG) protein databases to identify functionally annotated proteins with the most similar sequences. The nr annotations, Gene Ontology (GO) annotations, and functional classification of the transcripts were determined using Blast2GO [34,35] and WEGO [36]. Pearson’s correlation coefficient was used to evaluate the correlations between the transcripts and each GO term and KEGG biological pathway for the three transcriptomes.

### 4.5. Identification of Enriched GO Terms and KEGG Pathways

The hypergeometric test was used to identify the significantly enriched GO terms in the target gene groups (compared with the control) [37,38]. The following formula was used to assess significance: $p=1-\overset{m-1}{\underset{i=0}{\sum }}\frac{\left(\begin{array}{c} M\\ i \end{array}\right)\left(\begin{array}{c} N-M\\ n-i \end{array}\right)}{\left(\begin{array}{c} N\\ n \end{array}\right)}$, where *N* is the number of all of the genes annotated with a GO term, *n* is the number of DEGs in *N*, *M* is the number of genes that are annotated with a specific GO term, and m is the number of DEGs in *M*. The threshold for identifying significantly enriched GO terms was *p* < 0.005. In addition, to identify the enriched KEGG pathways, the hypergeometric test was used in a similar manner to determine the relative coverage of the annotated KEGG orthologous groups of pathways in the background. The threshold for identifying significantly enriched pathways was *p* < 0.005 [39].

### 4.6. Quantitative Real-Time PCR

Equal amounts of each RNA sample were used for the qRT-PCR and transcriptome sequencing analyses. The primer pairs (Supplementary Table S1) for the candidate genes were designed using Primer Premier 6.0. The total RNA was reverse transcribed using the Hifair^®^ II 1st Strand cDNA Synthesis SuperMix for qPCR (with gDNA digester plus) (Yeasen Biotechnology, Shanghai, China). The qRT-PCR analysis was performed using the CFX Connect Real-Time PCR instrument (Bio-Rad, Berkeley, CA, USA) and the Hieff UNICON^®^ Universal Blue qPCR SYBR Green Master Mix (Yeasen Biotechnology). The PCR program was as follows: 95 °C for 60 s, 40 cycles of 95 °C for 15 s, and 60 °C for 35 s. Relative gene expression levels were calculated according to the 2^−ΔΔCt^ method [40].

### 4.7. Statistical Analysis

All of the experiments were independently performed three times with each treatment replicated five times. The qRT-PCR analysis of each sample was replicated three times. Data were analyzed using SPSS 17.0 (SPSS Inc., Chicago, IL, USA) and Origin 8.0 (OriginLab, Northampton, MA, USA). A one-way analysis of variance (*t*-test) was completed to determine the significance of any differences among the treatments (*p* < 0.05).

## Figures and Tables

**Table 1:** Quality of the sequencing data for the CgWT0h and CgWT12h samples. Note: CgWT0h, *C. gloeosporioides* wild-type conidia; CgWT12h, *C. gloeosporioides* wild-type mature appressorium.

Sample	Data (bp)	Clean Reads	Q30 (%)	Total Mapping (%)	Uniquely Mapping (%)
CgWT0h	7,534,480,292	25,188,447	94.46	91.76	91.38
CgWT12h	6,803,953,388	22,770,082	94.81	93.11	92.85

**Table 2:** Upregulated and downregulated *C. gloeosporioides* genes between the conidia and mature appressorium.

Condition	ID	log_2_FC	*p*-Value	Annoation
CgWT0h-CgWT12h-up	EVM0007582	6.3931	8.68 × 10^−7^	Fum1, highly reducing polyketide synthase
	EVM0002071	5.3559	8.20 × 10^−5^	Acyl-CoA-binding protein
	EVM0007891	5.2385	9.51 × 10^−5^	AtmM, FAD-dependent monooxygenase
	EVM0001628	5.1897	1.13 × 10^−4^	Asl1, alkali-sensitive linkage protein
	EVM0011845	4.8421	2.75 × 10^−4^	FadD13, long-chain-fatty-acid-CoA ligase
	EVM0001548	4.4904	9.39 × 10^−4^	ApdG, acyl-CoA dehydrogenase
	EVM0011186	4.4468	7.35 × 10^−4^	Nrk1, nicotinamide riboside kinase
	EVM0005520	4.3341	8.93 × 10^−4^	Fkr-3, FK506-binding protein
	EVM0001282	4.1999	1.41 × 10^−3^	4-hydroxy-2-oxoglutarate aldolase
	EVM0006481	4.0481	2.77 × 10^−3^	Multifunctional cytochrome P450 monooxygenase
CgWT0h-CgWT12h-down	EVM0008257	5.6860	2.04 × 10^−5^	PelA, pectate trisaccharide-lyase
	EVM0008999	5.0929	1.84 × 10^−4^	Presilphiperfolan-8-beta-ol synthase
	EVM0001567	4.5656	1.06 × 10^−3^	DIT1, spore wall maturation protein
	EVM0009287	4.4408	1.40 × 10^−3^	Nmral1, NmrA-like family domain-containing protein
	EVM0007786	4.4165	1.33 × 10^−3^	Sodium transport ATPase 5
	EVM0005978	4.3315	1.66 × 10^−3^	Xyn1, endo-1,4-beta-xylanase
	EVM0006498	4.2992	1.67 × 10^−3^	SDH-4, L-sorbose-1-dehydrogenase
	EVM0013274	4.2895	1.75 × 10^−3^	Transporter
	EVM0001800	3.8492	5.65 × 10^−3^	Sulfoquinovose-1-dehydrogenase
	EVM0011157	3.8457	5.28 × 10^−3^	Multicopper oxidase

**Table 3:** Top 10 enriched KEGG pathways among the DEGs in the CgWT12h sample.

KEGG (KO) Term	No. Genes
Carbon metabolism	201
Biosynthesis of amino acids	198
Tryptophan metabolism	153
Glycine, serine and threonine metabolism	138
Fatty acid metabolism	130
Phenylpropanoid biosynthesis	126
Tyrosine metabolism	121
Arginine and proline metabolism	111
Glycerophospholipid metabolism	99
Carotenoid biosynthesis	96

## Data Availability

Data are contained within the article or Supplementary Material.

## Supplementary Materials

The following supporting information can be downloaded at: https://www.mdpi.com/article/10.3390/ijms24087411/s1.
