# Supplementary material for: Comparative Transcriptome Analysis Reveals the Effect of the DHN Melanin Biosynthesis Pathway on the Appressorium Turgor Pressure of the Poplar Anthracnose-Causing Fungus Colletotrichum gloeosporioides
Source: Int J Mol Sci. 2023 Apr 18;24(8):7411. doi: 10.3390/ijms24087411 (PMC10138971; doi:10.3390/ijms24087411)
Supplement: Supplementary file 1 [file ijms-24-07411-s001.zip › ijms-2288060-supplementary.pdf]

Table S1 Primer sequences

Primers	5'-3'
q-ALP1-F	GGATCAGGCGGCAGTAGTAT
q-ALP1-R	AAGCCAGTTCTGAAGACCGA
q-FadD13-F	ATCAAGGTCGTCACAGCAGA
q-FadD13-R	CAGATCTGCGCAATGAGACC
q-Aryl-alcohol dehydrogenase-F	TCTTGCGCATCATACCGTTG
q-Aryl-alcohol dehydrogenase-R	CCTGGTTCTTGGCTGCAATT
q-Crf1-F	ACTTCCCGTTCGAGCTTACA
q-Crf1-R	GGTAGAGCCGTTGAGAGGTT
q-Glycosyl hydrolases family 16-F	CCCACCTTCGTTGACAACTC
q-Glycosyl hydrolases family 16-R	AACCTCCCCACCAGTGTAAG
q-Alpha-glucosidase-F	CAAAGCCGACTCTGATTCCG
q-Alpha-glucosidase-R	AGCGCTCTCCAGGTAATGTT
q-Bms1-F	ATAGCAGCAACAGGGACAGT
q-Bms1-R	CTTGGACAGTGCACGTTTGA
q-ESF1-F	AGGAAAAGGAAGAGGAGCCC
q-ESF1-R	TCGCCCTCCTTCTTTTCCTT
q-TFIIIB-F	CAGAAGGAAGCAGACATGGC
q-TFIIIB-R	CAGCCTTCTTCTTCGCCTTC
q-YheN-F	CGGTATTTCCTTGCGCATCA
q-YheN-R	TCACATCCGAGGACAACGAA
q-Glycerol-3-phosphate dehydrogenase-F	GCGTCATCTTCGTTCTTCCC
q-Glycerol-3-phosphate dehydrogenase-R	TCTGTGAGATGCGGAGGTTT
q-4-hydroxy-2-oxoglutarate aldolase-F	CAACAGTAGCCTTCTTCCGC
q-4-hydroxy-2-oxoglutarate aldolase-R	ATGAGTTGTCTTTCGGCGTG

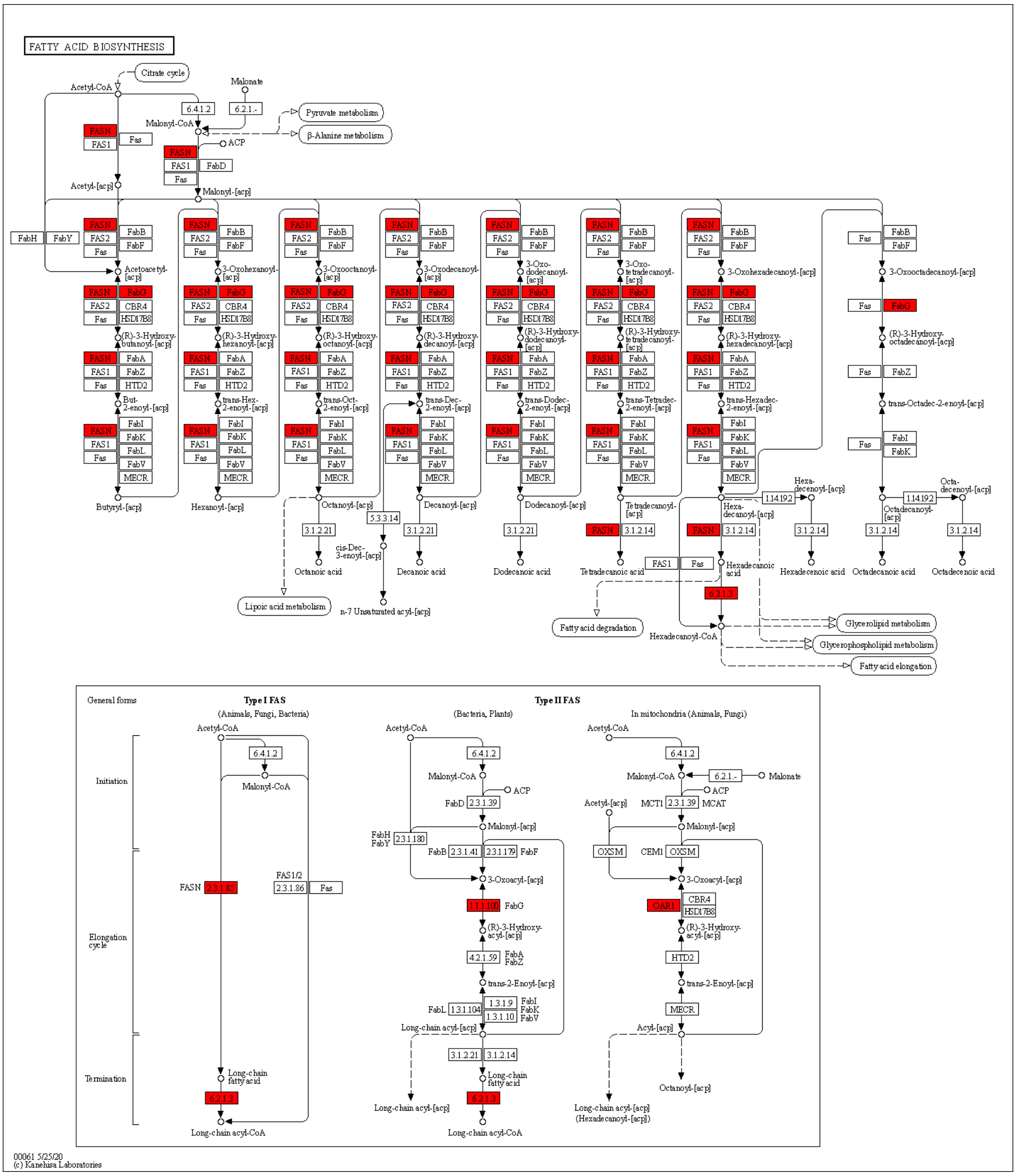

Figure S1 Fatty acid biosynthesis (ko00061)

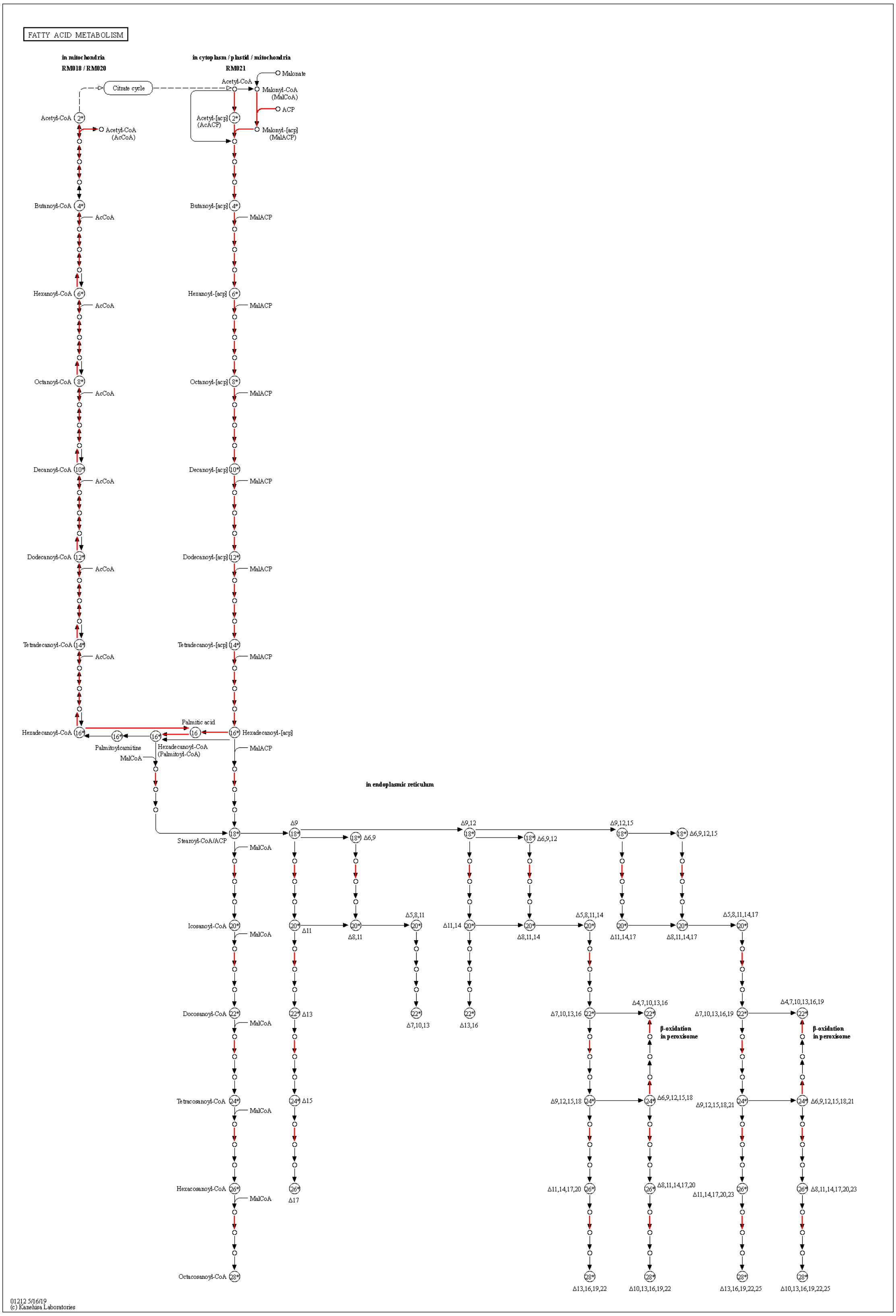

Figure S2 Fatty acid metabolism (ko01212)

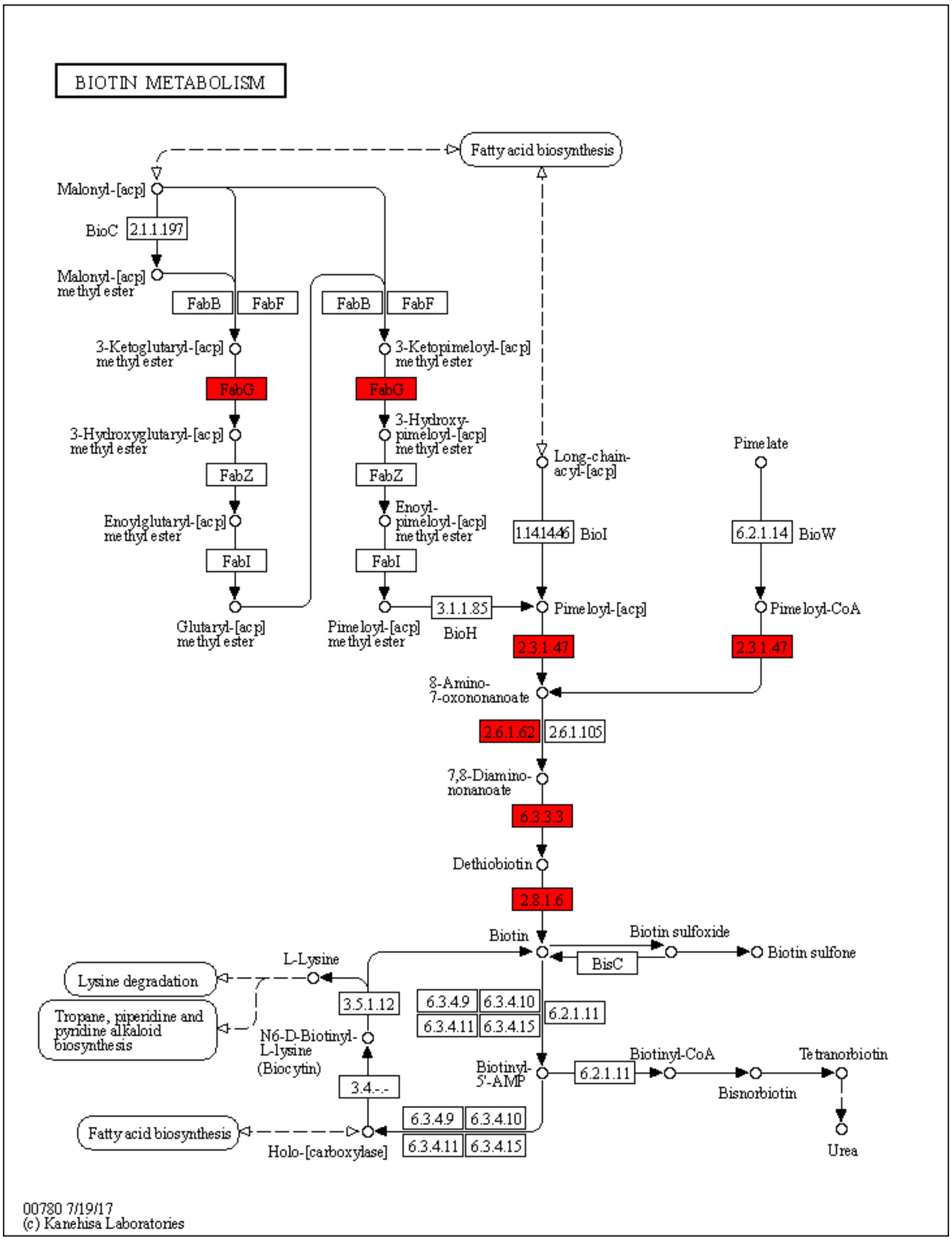

Figure S3 Biotin metabolism (ko00780)
